# Ovarian Cancer Targeted Theranostics

**DOI:** 10.3389/fonc.2019.01537

**Published:** 2020-01-21

**Authors:** Sridhar Nimmagadda, Marie-France Penet

**Affiliations:** ^1^Division of Nuclear Medicine and Molecular Imaging, The Russell H. Morgan Department of Radiology and Radiological Science, The Johns Hopkins University School of Medicine, Baltimore, MD, United States; ^2^Sidney Kimmel Comprehensive Cancer Center, The Johns Hopkins University School of Medicine, Baltimore, MD, United States; ^3^Division of Cancer Imaging Research, The Russell H. Morgan Department of Radiology and Radiological Science, The Johns Hopkins University School of Medicine, Baltimore, MD, United States

**Keywords:** ovarian cancer, diagnosis and therapy, molecular imaging, theranostic, targeted therapy

## Abstract

Ovarian cancer is a leading cause of death from gynecological malignancies. Although the prognosis is quite favorable if detected at an early stage, the vast majority of cases are diagnosed at an advanced stage, when 5-year survival rates are only 30–40%. Most recurrent ovarian tumors are resistant to traditional therapies underscoring the need for new therapeutic options. Theranostic agents, that combine diagnostic and therapeutic capabilities, are being explored to better detect, diagnose and treat ovarian cancer. To minimize morbidity, improve survival rates, and eventually cure patients, new strategies are needed for early detection and for delivering specifically anticancer therapies to tumor sites. In this review we will discuss various molecular imaging modalities and targets that can be used for imaging, therapeutic and theranostic agent development for improved diagnosis and treatment of ovarian cancer.

## Introduction

Ovarian cancer is the leading cause of death from gynecological malignancies and ranks fifth as a cause of cancer-related deaths among women. Nearly 14,240 deaths and 22,280 new cases of ovarian cancer are reported in the US every year (1). The majority of ovarian cancer related deaths, similar to other cancers, are due to metastatic disease. Nearly 90% of ovarian cancers are of epithelial origin and demonstrate various subtypes, serous, endometrioid, clear cell and mucinous, and have unique signature biomarkers for classification, some of which could be used for targeting. Vast majority (~80%) of epithelial ovarian cancers are diagnosed at an advanced stage, with presentation of widely metastatic disease within the peritoneal cavity. The 5-year survival rate for extensive stage disease remains low at 30–40%, with limited therapeutic options.

Current first-line treatment of high-grade epithelial ovarian cancer involves a debulking surgery that is followed by combination chemotherapy consisting of carboplatin and paclitaxel. However, long-term results from those therapies have been disappointing (2). Cytoreductive surgery is used as therapeutic intervention, as well as for diagnosis and staging. The amount of residual disease following a debulking surgery is considered a prognostic factor of survival and the absence of macroscopic residual disease is associated with low recurrence. Overall survival of advanced stage ovarian cancer patients showed little improvement in the last 30 years despite progress made in surgery and therapy. New targeted strategies are urgently needed to minimize morbidity and mortality associated with ovarian cancer. Theranostic approaches that combine diagnostic imaging with therapy have been shown to improve patient survival in several difficult to treat advanced cancers, and could also be applied to ovarian cancer, a focus of this review (3–5).

Mainly due to the lack of effective biomarkers to detect an early stage disease, ovarian cancer is most frequently diagnosed at an advanced stage. In this review, we will focus on various molecular targets being used to develop imaging, therapeutic and theranostic agents. High and specific expression of a biologic target is paramount for cancer detection. Additionally, inter- and intra-tumoral target expression heterogeneity is one of the hallmarks of cancer and is poorly understood in the context of advanced stage disease. The theranostic concept relies on identifying appropriate molecular targets highly specific to the cancer cells and assessing their expression levels and distribution by imaging that can be subsequently used for guiding appropriate therapy. The effectiveness of the approach comes from using imaging to select the patient population who would most likely benefit from the therapeutic agent, thus minimizing any off target effects and toxicity. This process requires the development and optimization of ligands for use as imaging/contrast agents that bind to proteins overexpressed in ovarian cancers. Those molecularly-targeted imaging agents help determine the tumor location and heterogeneity in target expression. They also allow assessment of the dosage and timing of drug administration, and ultimately of tumor response to therapy. The imaging component can also act as a surrogate for the possible therapeutic agent that is likely to demonstrate similar chemical and *in vivo* pharmacokinetic properties. Imaging and therapeutic molecules that are chemically or biologically identical or agents that are not identical but have similar enough biodistribution can be used.

Currently, the most clinically used theranostic agents are primarily radiopharmaceuticals wherein a highly specific molecularly targeted and optimized ligand is used for chelating a radionuclide with imaging properties that can be readily swapped for a radionuclide with therapeutic properties (6). Radionuclide therapies also provide the benefits of bystander, crossfire and abscopal effects that could lead to sterilization of the tumor as a whole. With chemo- or targeted therapies only cells binding to the therapeutic agent are destroyed. In contrast, with therapeutic radionuclides, the emitted radiation particle path length is longer than several cell diameters, and cell death can be observed in multiple cells in the neighborhood of a cell with the accumulation of the targeted therapeutic radionuclide. Those effects provide an additional advantage to address the heterogeneity of the tumors over targeted therapies because cells and tissues not expressing the target and present in the particle path can still be impacted by the radiation. Bystander effects induced by radiation could also occur in cells that have not themselves been exposed to the radiation but have received a signal from a neighboring irradiated cell and behave as though it had been irradiated which leads to genomic instability and cell death (7, 8). Abscopal effect refers to an effect away from the target. It is an immune system rendered response to ionizing radiation by cancer cells that are located distant from the cancer cells with the accumulation of the therapeutic radionuclide or the irradiated site in the case of external beam radiotherapy (9). With the advent of immune checkpoint therapeutics that activate the immune system, therapies combining radionuclide therapy with immunotherapy have the potential to boost the abscopal response rates.

Other theranostic approaches being explored include the use of a single platform strategy such as near-infrared photoimmunotherapy (NIR-PIT) that incorporates therapeutic and diagnostic components in one entity (10). NIR-PIT is a target specific therapy involving an antibody conjugated to a photoabsorber that binds to target cells and causes cellular damage upon subsequent exposure to NIR light. The irradiation with NIR light at 690 nm causes cell membrane damage and necrotic cell death. The specificity of NIR-PIT comes from the specificity of the antibody, injected intravenously for tumor targeting, and the toxicity induced by the photosensitizer after exposure to NIR light (11). NIR-PIT induces phototoxic effects only when NIR irradiation and cell membrane binding are combined. Importantly, NIR-PIT does not require intracellular delivery of the therapeutic agent. The NIR emission of IR700 dye can also be used for non-invasive fluorescence detection to optimize the delivery of the theranostic irradiation.

Another strategy is targeted nanoparticle delivery to the ovarian tumors (12). Nanoparticles make it feasible to deliver multiple imaging and therapeutic components simultaneously to the cancer cells, and enable an on demand or environmentally responsive therapeutic release once a sufficient concentration of payload reaches the tumor.

We will discuss various imaging modalities and targets that could be used for imaging, therapeutic and theranostic agent development to diagnose and treat ovarian cancer, as listed in Table 1.

**Table 1:** Molecules, modalities and applications of ovarian cancer targeted theranostics.

**Molecular target**	**Imaging/Theranostic agent**	**Imaging method**	**Potential clinical application**	**References**
CA125	^89^Zr-DFO-mAb-B43.13	PET	Cancer detection	(13)
	^99^Tcm-MAb-B43.13	SPECT	Cancer detection	(14)
	Nanobubbles	US	Cancer detection and drug delivery	(15)
Folate receptor	Mirvetuximab soravtansine (IMGN853)	PET	Cancer detection	(16–18)
	^89^Zr-radiolabeled M9346A (parent mAb of IMGN853)	PET	Pre-screen cancer patients for IMGN853 treatment	(19, 20)
	Folate or folate analog (EC17) conjugated to FITC	Optical	Real-time surgical visualization of tumors for intraoperative staging and surgical resection	(21, 22)
	^64^Cu-labeled pyropheophorbide-folate conjugate	PET and optical	Detection and intraoperative guidance of cancer resection	(23)
	SPION-CDF-FA-PAMAM	MR	Cancer detection and treatment	(24)
	PLGA-RbCur-gadolinium complex	MR	Combination therapies	(12)
	Microbubbles loaded with paclitaxel and oxygen	US and US targeted MB destruction	Anti-cancer drugs and/or oxygen delivery for combination therapy	(25)
Her2	^89^Zr-trastuzumab	PET	Cancer detection	(26, 27)
	^89^Zr-pertuzumab	PET	Cancer detection	(28–30)
	^177^Lu or ^212^Pb radiolabeled trastuzumab	Targeted radiotherapy	Cancer therapy	(31–33)
	IR700DX-trastuzumab	NIR-PIT	Cancer detection and treatment	(11)
	EC1-GLuc-liposome	bioluminescence	Cancer detection and treatment	(34)
NaPi2b (SLC34A2)	^211^At MX35 F(ab′)2	Targeted radiotherapy	Cancer therapy	(35)
EGFR	Cetuximab- benzoporphyrin derivate conjugate	NIR-PIT	Cancer detection and treatment	(36)
KDR	Microbubbles	US	Cancer detection	(37)
GSA	GSA-IR700	NIR-PIT	Cancer detection and treatment	(38)
β-galactosidase	HMRef-βGal SPiDER-βGal	Optical	Laparotomic and endoscopic detection of tumor and metastases	(39, 40)

## Imaging Techniques Used for Ovarian Cancer Detection and Diagnosis

Currently, women with a clinical suspicion of ovarian cancer are assessed with pelvic examination, transvaginal ultrasound (TVUS), and serum biomarkers. However, these techniques have significant limitations in the accuracy of detection and characterization of ovarian malignancy. Staging of ovarian cancer is usually done with histology and computed tomography (CT) to decide treatment and surgical procedures. Ultrasound (US) imaging is frequently used in the diagnosis of ovarian cancer (41, 42). The reported accuracies for distinguishing malignant from benign tumors by US is 65–94%, 35–88%, and 48–99% for gray-scale, color Doppler flow imaging, and Doppler arterial resistance measurements, respectively (43). In a meta analysis Kinkel et al. showed that sonographic techniques combining gray scale morphologic assessment with tumor vascularity imaging information are significantly better in characterizing ovarian lesions compared to individual measurements alone. The Q^*^ point (and 95% CI) for combined techniques was 0.92 (0.87, 0.96) vs. 0.85 (0.83, 0.88), 0.82 (0.78, 0.86), and 0.73 (0.58, 0.87) for morphology, Doppler US and color Doppler flow imaging, respectively (43). Q^*^ values correspond to the point on the summary receiver operating characteristic (ROC) curve where sensitivity and specificity were equal.

To further improve ovarian cancer detection by US, different non-targeted and targeted contrast agents have been developed and explored. Application of microbubbles (MB) as a contrast agent to improve the detection of early stage ovarian malignancies has been explored in preclinical (44), and clinical studies (45). Intravenous injection of non-targeted perflutren MB contrast agent was used to image 26 benign masses and 10 malignancies, to acquire the following parameters: presence of contrast enhancement, time to peak enhancement, peak contrast enhancement, half wash-out time, and area under the enhancement curve (AUC). The study showed that an AUC >787 s^−1^ was the most accurate diagnostic criterion for ovarian cancer with high sensitivity (100.0%) and specificity (96.2%). Other values that were found to be useful are the peak contrast enhancement >17.2 dB that showed 90.0% sensitivity and 98.3% specificity, and half wash-out time >41 s that 100.0% sensitivity and 92.3% specificity (45).

Optical imaging is being extensively used in preclinical settings and more recently in the clinic intraoperatively. Intraoperative fluorescence imaging could be applied to improve tumor staging and debulking in the course of cytoreductive surgery to improve prognosis. Multiple tumor-specific intraoperative fluorescence probes have been developed, and applied to ovarian cancer, both in preclinical (39), and clinical (21, 22) studies. Those clinical studies demonstrated the potential benefit of a systemically administered tumor-specific targeted fluorescent agent for intraoperative fluorescence imaging and in staging and debulking surgery for ovarian cancer.

Positron emission tomography (PET) imaging is highly sensitive and provides specific and quantitative information of a disease process given a molecularly targeted imaging agent is available. The most conspicuous radiotracer ^18^F-Fluorodeoxyglucose reports on upregulated glucose metabolism in tumors and is routinely used in the management of ovarian cancer for diagnosis, staging, and for monitoring tumor response to chemotherapy (46).

Due to cost and availability limitations, Magnetic resonance imaging (MRI) is not frequently used for ovarian cancer diagnosis. MRI and magnetic resonance spectroscopy (MRS) have been applied to describe the feasibility of effective cancer imaging to assess complex ovarian masses indeterminate on either palpation or ultrasonography. MRI diagnostic criteria of ovarian malignancies are usually based on morphology, thick septum, vegetations, ascites, lymphadenopathy, and contrast characteristics. MRI can also be used for functional and metabolic imaging *via* dynamic contrast enhanced (DCE)-MRI, diffusion weighted imaging (DWI) and MRS, in addition to gaining the anatomic information. DCE-MRI is used to characterize ovarian cancer noninvasively (47), and to distinguish benign from malignant lesions on the basis of differences in contrast agent distribution and uptake that are manifested by neoangiogenesis induced microcirculation (48). Carter et al., showed that quantitative parameters from DCE-MRI and T_2_ mapping could be used to differentiate benign (*n* = 22) from malignant (*n* = 12) ovarian masses (49). MRS has been proved useful in diagnosis, grading and treatment planning of brain and prostate cancer. However, studies using MRS for female pelvic lesions are limited, due to technical issues that arise from susceptibility to motion artifacts. The few reported clinical studies have shown the feasibility of using single voxel spectroscopy to differentiate benign from malignant lesions by measuring total choline (tCho) levels (50, 51). Booth et al., have shown that MRI at 3T can be used to stage ovarian cancer with an accuracy comparable to that obtained with surgical staging (52). 3D MRSI of ovarian masses can also be performed at 3T to allow an accurate spatially resolved analysis of ovarian cancer (53). There are however some limitations associated with using MRS alone. tCho in primary and metastatic ovarian tumors can be quantified using ^1^H MRS, although a high rate of failure has been observed (51). Moreover, tCho was detected at 3T in both benign and malignant gynecological neoplasms (52), highlighting the importance of combining multiple MR techniques in order to improve tumor detection and staging.

## Molecular Targets Used for Ovarian Cancer Detection and Therapy

### CA125

The overexpression of MUC16/cancer antigen 125 (CA125) is one of the hallmarks of high-grade serous ovarian cancer (HGSOC) (54). CA125, a mucin-type-*O*-linked glycoprotein, is expressed as a membrane bound protein at the ovarian cancer cell surface. It is also released in soluble form in bodily fluids. CA125 level in serum, quantified *via* immunoassays, is one of the most extensively studied biomarker for ovarian cancer. CA125 measurements are applied in clinical management of ovarian cancer in different situations including early detection, disease monitoring, early prediction of outcome, tumor status after completion of chemotherapy, and early detection of recurrence (55, 56).

**Figure 1:**
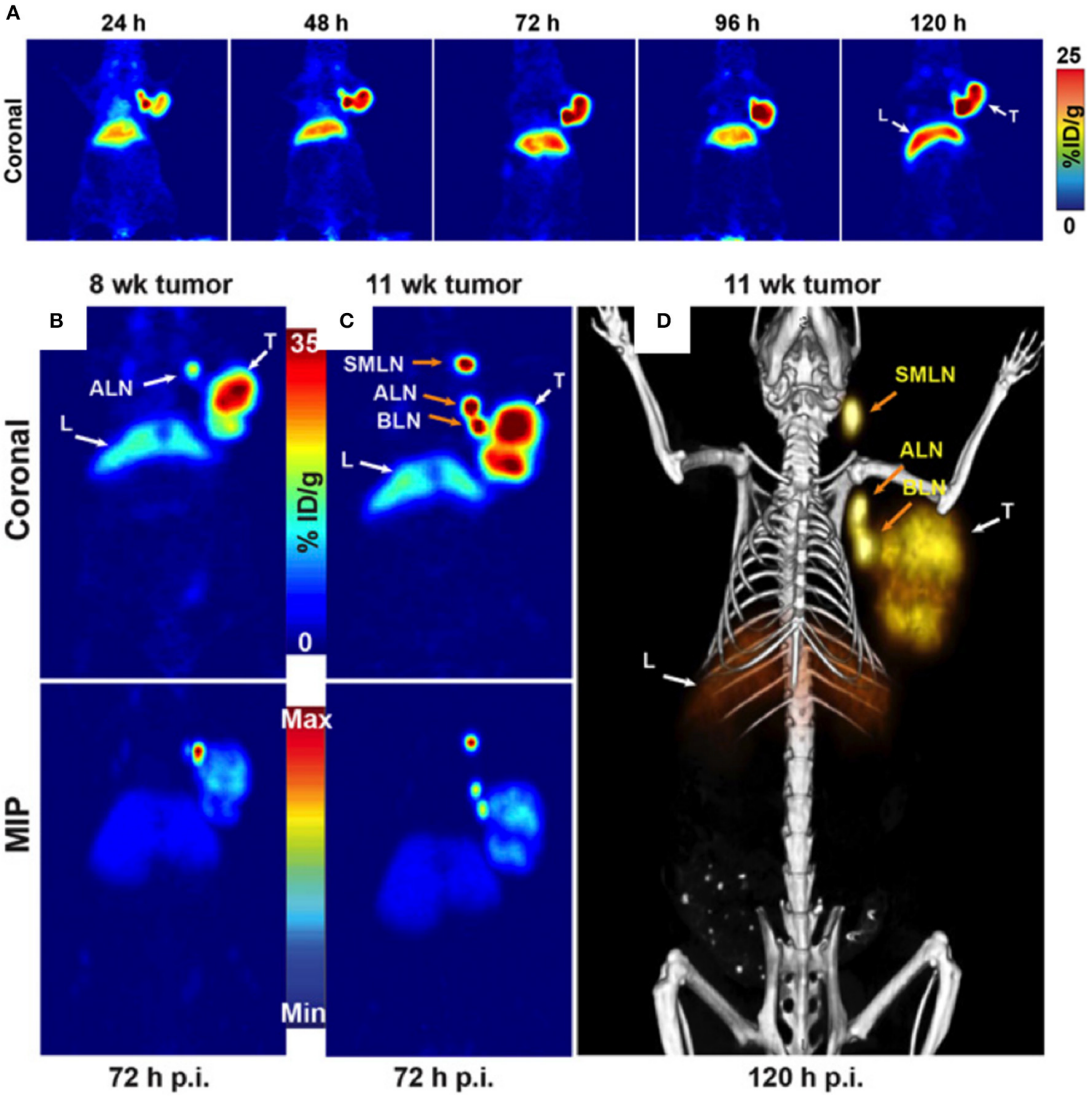
**(A)** Serial PET images of an athymic nude mouse bearing a CA125-positive OVCAR3 xenograft after tail vein injection of ^89^Zr-DFO-mAb-B43.13 after (10.2–12.0 MBq). Coronal planar images intersect the middle of the tumor. **(B)** Coronal (top) and maximum-intensity projection (MIP; bottom) PET image obtained 72 h after administration of ^89^Zr-DFO-mAb-B43.13 to a mouse 8 week post implantation of an OVCAR3 xenograft on the right shoulder. **(C)** Coronal (top) and MIP (bottom) PET images obtained 72 h after administration of ^89^Zr-DFO-mAb-B43.13 to the same mouse 11 week post tumor implantation. **(D)** PET/CT image obtained 120 h after administration of ^89^Zr-DFO-mAb-B43.13 to the same mouse 11 week post tumor implantation. ALN, axillary LNs; BLN, brachial LN; L, liver; p.i., post-injection; SML, submandibular LN; T, tumor. Adapted with permission from Sharma et al. (13).

Lymph node involvement is observed in 75% of ovarian cancer cases of late stage disease. Serum-based biomarkers, such as CA125, despite their routine use and low cost, fail to accurately pinpoint the lymph nodes involved and the site of recurrence. PET probes targeting CA125 could be valuable tools in the management of ovarian cancer for whole body visualization and quantification of CA125. Antibodies that bind to CA125 have been reported and can be readily converted into PET imaging agents by conjugating imaging radionuclides with suitable half-life such as zirconium-89 (3.27 days). Taking advantage of the available antibodies, Sharma et al., have demonstrated that a Zr-89 labeled anti-CA125 murine antibody B43.13 (57) (or ^89^Zr-DFO-mAb-B43.13) could be used for clear delineation of CA125 positive human tumor xenografts from negative tumors in mouse models (13) (Figure 1). CA125 positive tumors were detected as early as 24 h after radiotracer injection. In mouse models with lymph node involvement, the authors also observed high levels of ^89^Zr-DFO-mAb-B43.13 concentrations in lymph nodes by PET imaging. That increased uptake observed was cancer specific and confirmed by pathological validation of metastatic disease in excised samples. PET negative lymph nodes were found to be free of metastatic disease further validating the specificity of the radiotracer. Collectively, these data show the potential for development of a theranostic variant for detecting and targeting CA125 positive ovarian cancer as one could readily generate a radiotherapeutic version of the antibody. The pharmacokinetics and radiation dosimetry of B43.13 labeled with ^99m^Tc, a SPECT radionuclide, have been reported previously (14). Additional clinical studies with B43.13 as an imaging agent have not yet been reported but B43.13 is being tested in combination with other therapeutics in several clinical trials (58).

CA125 has also been shown as a potential target for contrast enhanced ultrasound imaging. CA125-targeted echogenic lipid and surfactant-stabilized nanobubbles were used in a mouse model to image CA125 positive OVCAR3 tumor with a standard clinical contrast harmonic ultrasound (15). An enhanced tumor accumulation of nanobubbles, higher peak ultrasound signal intensity and slower wash out rates were observed in OVCAR3 tumors compared to CA125 negative SKOV3 tumors. The CA125 binding nanobubbles also showed increased tumor retention and prolonged echogenicity compared to untargeted nanobubbles. The study results suggest that CA125 antibody-conjugated nanobubble-based ultrasound molecular imaging could potentially improve diagnosis of CA125 positive ovarian cancer (15).

### Folate Receptor

Another target that is of significant interest in ovarian cancer is folate receptor-α (FRα), a glycosylphosphatidylinositol (GPI)-anchored membrane glycoprotein with a high affinity to folic acid. Nearly 90% of HGSOC overexpress FRα (59). Expression of FRα in normal tissues is negligible, thus providing an opportunity for FRα specific delivery of theranostics to the tumor. Folic acid binding results in internalization and sequestration of the bound conjugates. Taking advantage of that phenomenon, folate has been used to develop PET, SPECT, and fluorescence-based imaging agents that yield highly resolved images of FRα positive tumors in preclinical models (60).

A variety of folate-derived conjugates have been developed as PET imaging agents incorporating most clinically used radionuclides F-18 and Ga-68 (16). The overexpression of FRα in cancer has also led to the development of a variety of FR targeted therapeutics including antibody drug conjugates. One such agent is mirvetuximab soravtansine (IMGN853), an FRα-targeting humanized monoclonal antibody-drug conjugate. IMGN853 is being tested in several clinical trials in cancer patients, including platinum resistant ovarian cancer patients (17, 18). Selection of patients for those therapies currently relies on immunohistochemistry (IHC) analysis of archived biopsies. To improve patient selection and therapeutic intervention, two studies developed a ^89^Zr-radiolabeled version of M9346A as a radiotracer for FRα detection. M9346A is the parent antibody of IMGN853 (19, 20). Evaluation of ^89^Zr-M9346A uptake in patient derived xenograft models of triple negative breast cancer and ovarian cancer with graded expression levels of FRα showed target specificity, sensitivity and correlated with treatment response to antibody drug conjugate. Those studies portend the potential of ^89^Zr-M9346A PET as a theranostic tool to pre-screen ovarian or other cancer patients for IMGN853 treatment.

**Figure 2:**
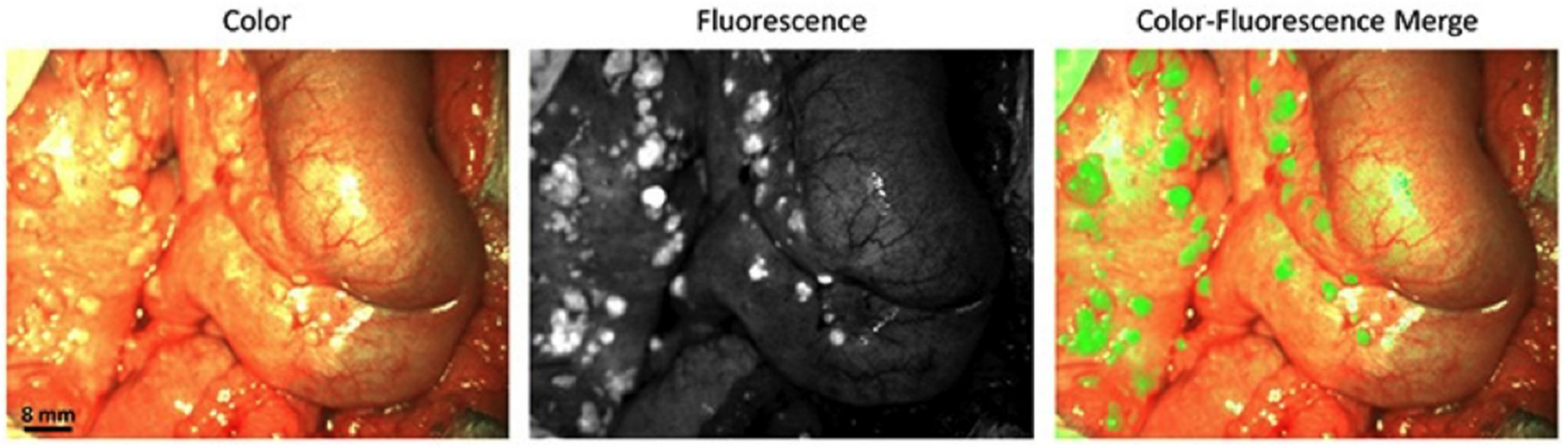
Biopsies of lesions found histologically to be metastases of serous adenocarcinoma. In total, 57 fluorescent lesions that were identified during surgery were resected. Of these resected lesions 44 (77%) appeared to be malignant on histopathology. Seven (16%) of these 44 lesions were not detected by visual inspection with the naked eye or palpation either because they appeared benign or because they were missed during inspection due to small size (<10 mm) and flat nature. Adapted with permission from Tummers et al. (22).

FRα can also be used as a target for optical imaging. Folate conjugated with fluorescein isothiocyanate was used for real-time visualization of tumors in patients for suspected ovarian cancer and undergoing laparotomy. In that study, fluorescence was detected intraoperatively in all patients with FRα positive malignant tumors but not in FRα negative malignant tumors or benign tumors, facilitating fluorescence guided surgical resection of tumor deposits (21). Such agents could have direct impact on patient survival by facilitating improved intraoperative staging and surgical resection, as targeted probes improve specificity and sensitivity of cancer detection during surgery. These results were later confirmed in another study (22) where an FRα-targeting agent, EC17, was intravenously injected to ovarian cancer patients 2–3 h before surgery. EC17 consisted of a folate analog conjugated to 5-fluorescein isothiocyanate (FITC). Using fluorescence imaging ovarian cancer metastases located on the intestine and mesentery could be visualized in those patients (Figure 2). As a next step, the number of lesions and positive margins detected with fluorescence were measured, following which correlation was assessed between the fluorescent signal, the presence of a malignant lesion, and the FRα status. Fluorescence imaging detected 77% of lesions that appeared malignant on histopathology in ovarian cancer patients and 16% of those were not detected with inspection/palpation. A correlation between fluorescence and FRα- and tumor status was demonstrated by histopathology. In spite of a clear fluorescent signal produced by EC17 in ovarian cancer tissue, false-positives were observed due to the normal tissue expression of FRα, or auto-fluorescence signal from collagen and false negatives were linked to inadequate penetration depth of the fluorescence technology.

Those advances have also lead to the development of dual- and multi-modality agents. Liu et al., developed a multimodal PET and optical FRα-targeted agent for ovarian cancer imaging. The agent, ^64^Cu-labeled pyropheophorbide-folate conjugate, showed selective uptake in metastatic ovarian cancer deposits of <1 mm size that could assist in non-invasive tumor detection as well as in intraoperative guidance of cancer resection (23).

MRI has also the potential to be used to visualize delivery of FR targeting theranostic agent. Luong et al. developed a polyvalent theranostic nanocarrier consisting of a superparamagnetic iron oxide nanoparticle (SPION) core, loaded with a highly potent anticancer agent, 3,4-difluorobenzylidene-curcumin (CDF) and decorated with folic acid-polyamidoamine dendrimers (FA-PAMAM) (24). *In vitro*, those nanoparticles exhibited a high MR contrast and anticancer activity in ovarian (SKOV3) and cervical (HeLa) cancer cells that are known to overexpress FR. The intracellular accumulation and therapeutic effects were more pronounced with targeted particles compared to non-targeted ones. These studies showed the ability of multivalent theranostic nanoparticles for simultaneous imaging and therapy in cancer cells and will have to be confirmed *in vivo*.

Combination therapies can improve treatment efficacy. Novel dual therapy nanoparticles such as poly (lactic-co-glycolic acid) (PLGA) nanoparticles that simultaneously deliver a boron-curcumin complex (RbCur) and an amphiphilic gadolinium complex into tumor cells are being developed (12). Those nanoparticles combined boron and gadolinium neutron capture therapy with anti-proliferative effects of curcumin. The presence of gadolinium makes the nanoparticles visible to MRI. These nanoparticles were tested *in vitro* on ovarian cancer IGROV-1 cells with FR targeting. In those studies, an effective synergic activity was described when neutron treatment was combined with and curcumin cytotoxicity (12). The authors showed that the presence of curcumin before and during neutron exposure leads to increased cell mortality and significantly decreased proliferation of the surviving cells resulting in improved treatment outcome compared to gadolinium neutron capture therapy used alone (12).

Microbubbles (MBs) have the potential to deliver anti-cancer drugs and/or oxygen for combination therapy in addition to being used as contrast enhancement agents for ultrasound imaging (25). MBs have a core-shell structure and can effectively encapsulate anti-cancer drugs. The US targeted MB destruction (UTMD) technique has been applied to increase drug delivery to the tumors to improve therapeutic effect. Under ultrasound pulses, MBs undergo stable and inertial acoustic cavitations that induce a variety of dynamic processes leading to cell membrane disruption and facilitating intracellular uptake of drugs (25). When MBs are exposed to an US field, the mechanical wave causes them to cavitate. Cavitation is a broad term for US-induced oscillation, and collapse of bubbles (61). For drug delivery, the US parameters induce mechanical effects that also have the potential of enhancing the antitumor efficacy of drugs by increasing microvessel permeability, enhancing drug penetration through the interstitial space, and increasing tumor cell drug uptake (61). Enhanced therapeutic efficacy was demonstrated *in vitro* using US-targeted MB destruction with delivery of paclitaxel and oxygen (25). Ligands can be conjugated to the surface of the drug-loaded MBs to enhance cancer cell selectivity, as shown by Luo et al. who developed oxygen-paclitaxel loaded lipid MBs specifically targeted to FR expressing cells, demonstrating therapeutic efficiency in ovarian cancer xenograft models (62).

### HER2

The EGF receptor (EGFR) (or HER) proto-oncogene family consists of four transmembrane tyrosine kinase receptors (i.e., EGFR, ErbB2, ErbB3, and ErbB4) that play a role in cancer pathogenesis and has been described as key therapeutic target in many types of cancer, including ovarian cancer (63).

HER2 (or Erbb2) is a 185 KDa transmembrane glycoprotein known to be overexpressed in a variety of cancers including breast, ovarian, cervical, colon, endometrial, esophageal, lung, and pancreatic cancers (64). HER2 overexpression may confer a selective growth advantage to the tumor cells making it one of the most important biomarkers for guiding therapy. HER2 status determined by IHC and fluorescence *in situ* hybridization has been used to guide and predict the efficacy of anti-HER2 therapy (65). In ovarian cancer, HER2 overexpression has been reported as highly variable. A broad range of HER2 expression frequencies has been found based on IHC results, from 0 to 100% with an average frequency equal to 40% among malignant ovarian tumors across all studies (63). HER2 overexpression contributes to poor survival, and patients with HER2 positive tumors are treated with trastuzumab, a monoclonal antibody targeting HER2 that received FDA approval.

Because of the significant interest in quantifying changes in HER2 expression non-invasively, several antibodies and antibody fragments including affibodies and nanobodies have been developed as imaging agents. Those agents were then used to quantify HER2 expression non-invasively and to assess HER2 positive tumor response to therapy. Radiolabeled trastuzumab, ^89^Zr-trastuzumab, has demonstrated HER2 specific uptake in patients with metastatic breast cancer (26). Similarly, ^89^Zr-pertuzumab, another HER2 targeted antibody that inhibits dimerization of HER2, has shown promise in detecting HER2 positive tumors in preclinical models and in patients with metastatic disease (28–30). The optimal image contrast times for those intact antibodies are in days, which could potentially limit their routine use in the clinic. Therefore, varieties of agents that would allow image acquisition within hours after injection have been investigated to improve clinical use. For example, affibody and nanobody molecules labeled with a variety of radionuclides including ^18^F, ^68^Ga, and ^111^In have shown promise in detecting HER2 positive ovarian tumors within hours after the radiotracer injection (66, 67). Such agents could be used to pre-screen patients for HER2 targeted therapies and for real-time assessment of tumor response to therapy.

Imaging is also uniquely positioned to inform on the drug activity in real-time in the tumor. In patients, multiple mechanisms contribute to trastuzumab resistance and imaging has the potential to relate drug exposure at the tumor to response to therapy. In a study by Gebhart et al. radiolabeled trastuzumab was investigated in patients for non-invasive quantification of HER2 expression (27). Nearly one third of the breast cancer patients with tumors expressing HER2, as confirmed by IHC, showed little or no uptake of ^89^Zr-trastuzumab across their metastases. Those data suggest that penetration of a drug, in this case ^89^Zr-trastuzumab, into tumor tissue does not solely rely on target presence and that molecular imaging could provide insights into tumor response to therapy. Although those data were acquired in breast cancer patients, the results could have implications for all solid tumors, including ovarian cancers.

Ovarian cancer spreads through the intraperitoneal cavity contributing to significant morbidity and mortality. Low toxicity treatments for intraperitoneal disease are few and an unmet medical need. Intraperitoneal chemotherapy has improved survival but it is not a standard option and carries life-threatening toxicity (68). Targeted radiopharmaceutical therapy delivers the radiation directly to the target expressing cancer cells thus enhancing efficacy and limiting toxicity. Such radiopharmaceutical therapies are often delivered using α- and β-emitting radionuclides. Those radiotherapeutics provide multiple advantages including cross-fire effect and abscopal response that is not generally observed with conventional systemic therapies (9). The overexpression of HER2 and high selectivity of trastuzumab have been exploited to develop radioimmunotherapy. Trastuzumab radiolabeled with α- and β-emitting radionuclides have been investigated for the treatment of disseminated peritoneal disease and tumors with HER2 expression (31). The β-emitting ^177^Lu radiolabeled transtuzumab is being investigated in patients providing a theranostic approach to HER2 overexpressing cancers (32). Similarly, initial studies with β-emitting radioimmunotherapeutics of TAG-72, tumor-associated glycoprotein 72, for treating ovarian cancer have shown promise (69–71). However, the failure of anti-MUC1 HMFG1 antibodies conjugated with ^90^Y, a β-emitter, to improve survival in patients with intraperitoneal disease in Phase-III trials have prompted further investigation of α-particle therapies in ovarian cancer patients (72).

α-emitter therapy has been gaining attention for treating ovarian cancer with intraperitoneal dissemination. α-particles are highly suited for targeting single cells or small tumor clusters as they have a short range in tissues (< 100 μM) with high linear energy transfer that deposits a localized irradiation generating highly cytotoxic double strand breaks in the DNA (73). Intraperitoneal radioimmunotherapy using ^212^Pb conjugated to trastuzumab, an α-emitter, in a first-in-human study was found to be safe with patients showing a trend of decreasing tumor growth and blood-based biomarkers with increasing administered radioactivity (33). In another study Hallqvist et al., investigated intraperitoneal α-particle therapy using MX35, the antigen-binding fragments-F(ab′)2-of a mouse monoclonal antibody, conjugated with α-emitter ^211^At in epithelial ovarian cancer patients (35). MX35 F(ab′)2 fragment targets the cell surface glycoprotein NaPi2b (SLC34A2) that is expressed on more than 90% of human epithelial ovarian cancers (74). Long-term follow up of those patients showed no apparent signs of radiotoxicity and no decreased tolerance to relapse therapy, thus paving the way for the use of α-particle therapy in ovarian cancers.

Ovarian cancer is a great candidate for NIR-PIT as the light can be applied during cytoreductive surgery. NIR-PIT has been shown to induce effective cell killing of HER2 expressing SKOV3 cells, in subcutaneous and disseminated peritoneal ovarian cancer preclinical models (11). The antibody-photosensitizer conjugate consisted of trastuzumab and IR700DX. The antitumor effect was observed in both models after repeated light exposure, highlighting the potential role of NIR-PIT to treat disseminated peritoneal tumors (11).

Liposomes have been described in multiple studies as effective targeted drug delivery systems for cancer therapy. In the context of ovarian cancer, Han et al., have developed a liposome conjugated with a recombinant protein, EC1-GLuc, fusion of EC1 peptide, an artificial ligand of HER2, with *Gaussia* luciferase (GLuc) for bioluminescent imaging (34). This EC1-GLuc-liposome could be an effective theranostic system for HER2-overexpressing metastatic ovarian carcinoma by combining targeted imaging to drug delivery. *In vitro* experiments revealed selective targeting and internalization of the EC1-GLuc-liposome into HER2-overexpressing SKOV3 cells. To assess the intracellular delivery, a cell-impermeable fluorescence dye (HPTS) was encapsulated in EC1-GLuc-liposome and delivered into SKOV3 cells. *In vivo*, EC1-GLuc-liposomes targeted and delivered HPTS to metastatic SKOV3 tumors, as shown by bioluminescence imaging (34).

### EGFR

Epidermal growth factor receptor (EGFR, ErbB1, HER1) has been described as overexpressed in ovarian epithelial cancer of all histologic subtypes. IHC studies reported highly variable levels of EGFR expression among malignant ovarian tumors with results ranging from 4 to 100% of ovarian carcinomas expressing EGFR, and an average frequency of 48% across all studies (63). Leveraging that expression, NIR-PIT targeting EGFR has been applied to treat primary tumors as well as to target metastases. Residual micrometastases are difficult to detect by current imaging techniques and escape standard treatments. Ovarian cancer metastases spread by hematogenous and lymphatic routes and also through peritoneal dissemination. Removal of peritoneal metastases have been shown to improve overall survival in ovarian cancer patients, however, diffuse peritoneal dissemination often consists of a large number of unresectable sub-millimeter lesions that contribute to disease recurrence. To monitor and treat disseminated micrometastases, Spring et al., developed an EGFR targeting dual function activatable immunoconjugate (36) and demonstrated the efficacy of activatable PIT in an OVCAR5 peritoneal micrometastases model (36). That strategy allowed enhanced contrast imaging and selective delivery of therapy to micrometastases while decreasing background fluorescence and toxicity to vital tissues (36). In those studies, a benzoporphyrin derivate (BPD) was used as NIR photoactivatable cytotoxic chromophore. To achieve optimized tumor specificity and quenching, seven BPD conjugates were combined to EGFR targeting cetuximab antibody (Cet-BPD). Cet-BPD conjugates were trafficked to lysosomes through EGFR internalization, and degradation pathway. Intracellular release and dequenching of BPD led to activation of fluorescence emission resulting in phototoxicity. Furthermore, use of a fluorescence micro-endoscope allowed quantification of *in vivo* pharmacokinetics of the immunoconjugate and monitoring of metastatic burden reduction without the need of surgery and with a reduced non-specific phototoxicity, demonstrating the potential impact of the approach for metastatic disease.

### New Directions

**Figure 3:**
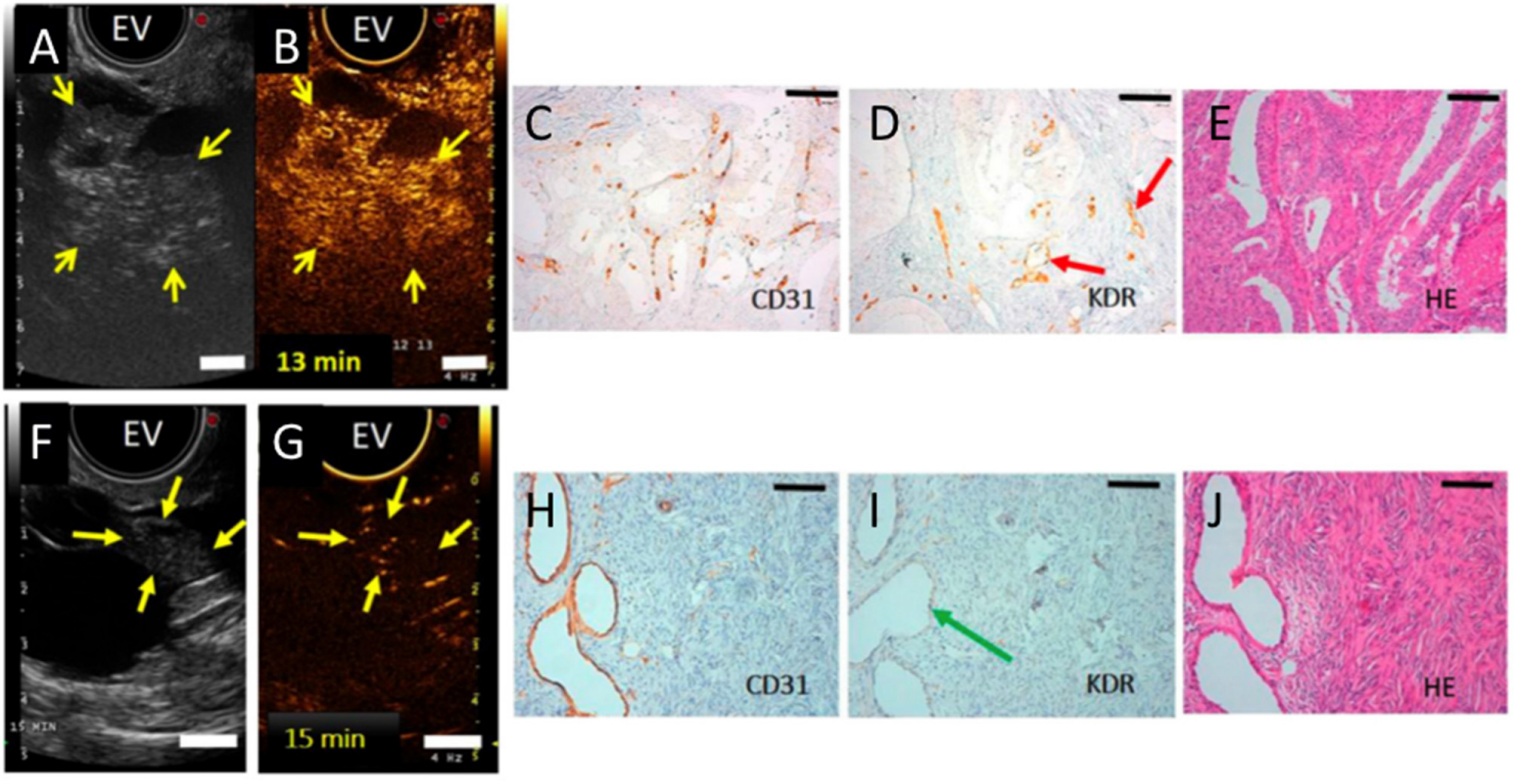
**(A)** Transverse endovaginal (EV) low mechanical index reference B-mode ultrasound image of the right ovary in a 50-year-old woman showing a 5.2-cm cystic and solid lesion (yellow arrows point to solid portion). **(B)** 13 min post iv injection of KDR-targeted contrast MBs (MBKDR), strong imaging signal is seen in the solid portion of the lesion (yellow arrows) on contrast mode ultrasound molecular image. **(C,D)** IHC performed on adjacent tissue sections demonstrating strong KDR expression on tumor-associated neovasculature (CD31+) (red arrows). **(E)** Histology showing endometrioid carcinoma. **(F)** Transverse endovaginal low mechanical index reference B-mode ultrasound image of right ovary showing a 4.8-cm cystic and solid ovarian lesion (yellow arrows point to solid portion) in a 65-year-old woman. **(G)** 15 min post iv injection of MBKDR, only minimal background signal is seen in 1.3-cm solid part of the lesion (yellow arrows) on contrast mode ultrasound molecular imaging. **(H,I)** IHC demonstrating minimal KDR expression (green arrow) on CD31+ vasculature. **(J)** Histology showing benign serous cystadenofibroma. Adapted with permission from Willmann et al. (37).

Several new targets and approaches are being evaluated for ovarian cancer targeting with an emphasis on improving detection and applications in image-guided surgery. A first-in-human clinical trial using ultrasound molecular imaging in patients with breast and ovarian lesions was recently performed (37). In that study, a kinase insert domain receptor [KDR] targeted clinical-grade microbubble contrast agent [MBKDR] was used. KDR is one of the key regulators of neoangiogenesis in cancer and angiogenesis plays a crucial role in the progression of ovarian cancer and metastases (75). As a phase I, the aim of the study was to assess safety and quantify KDR expression using the gold standard IHC. Patients with focal ovarian or breast lesions were intravenously injected with MBKDR, followed with ultrasound molecular imaging and then underwent surgical resection of the lesions. Those resected tissues were immunostained for CD31 and KDR. Results from those studies showed that MBKDR was well tolerated, and that KDR expression on IHC correlated with US imaging signal in 85% of malignant ovarian lesions, as shown in Figure 3. A robust KDR-targeted signal was observed in 77% of malignant ovarian lesions and absence of signal was noted in 78% of benign lesions (37).

**Figure 4:**
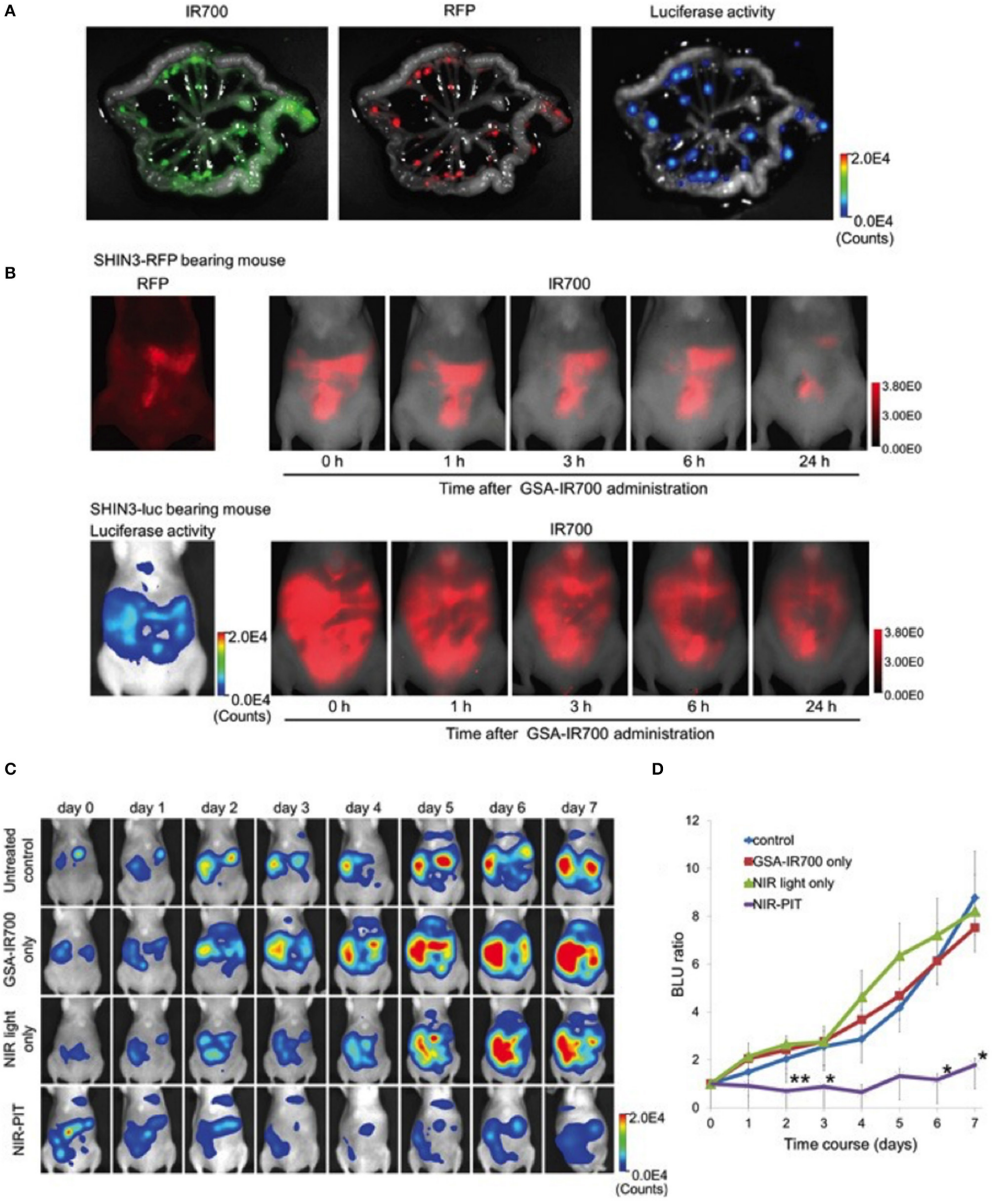
**(A)** IR700, RFP fluorescence, and luciferase activity in extracted mesentery from a SHIN3-luc-RFP tumor bearing mouse 3 h after i.p. injection of GSA-IR700. IR700 fluorescent signal and luciferase activity were mostly coincident with RFP positive foci. **(B)** *in vivo* serial IR700 fluorescence images of SHIN3-RFP (upper row) and SHIN3-luc (lower row) of tumor-bearing mice after administration of GSA-IR700. The distribution of GSA-IR700 correlated with the fluorescence of RFP or luciferase activity without evident accumulation in other organs up to 6 h after GSA-IR700 administration. **(C)** BLI of SHIN3-luc tumor bearing mice was obtained every day up to day 7. Luciferase activity decreased only in the NIR-PIT group. **(D)** Quantitative analysis of BLU ratio. Significant suppression of increment of BLU ratio was seen in the NIR-PIT group compared to other groups. Adapted with permission from Harada et al. (38).

NIR-PIT studies often use antibody as targeting moiety. A non-antibody derived NIR-PIT agent was developed by Harada et al., and tested in a disseminated ovarian cancer model (38). Galactosyl serum albumin (GSA) that is composed of galactose molecule conjugated to albumin *via* carboxyl groups was used. This construct can bind to beta-D-galactose receptor, a surface lectin, which is overexpressed in many cancers, including ovarian cancers. beta-D-galactose receptor is quickly internalized after binding to ligands. The agent was tested using SHIN3 cells that overexpress galactose receptor and produces diffuse peritoneal dissemination (38). GSA-IR700 probe accumulated specifically in the tumor, and repeated regimens of NIR-PIT improved the treatment efficacy by increasing the depth of GSA-IR700 delivery into tumor nodules (Figure 4). The study showed specific delivery of GSA-IR700 to the tumor (Figures 4A,B), and reduction of the metastatic burden after NIR-PIT (Figures 4C,D).

Nearly 50% of primary ovarian cancers show enhanced enzymatic activities of β-galactosidase compared to normal ovaries (76) and has been a focus of enzymatically activated fluorescence probe development to visualize ovarian cancer metastases (39, 40). Asanuma et al., developed a membrane-permeable HMRef-βGal allowing visualization of metastases of <1 mm in diameter in the peritoneal cavity after intraperitoneal administration of the fluorescence probe (40). More recently, a topically-sprayable and activatable fluorescent probe was developed to detect cancer, that would eliminate the need for an iv injection pre-surgery. After activation by the enzyme β-galactosidase, SPiDER-βGal can be retained within cells by anchoring to intracellular proteins. SPiDER-βGal was tested *in vitro* on different cancer cell lines and *ex vivo* on tumor tissues (39). SPiDER-βGal when compared to γGlu-HMRG, a probe activated by γ-glutamyltranspeptidase, demonstrated high sensitivity for detection of ovarian cancer metastases in the peritonium in a mouse model (39). SPiDER-βGal presented higher signal retention, and improved contrast of the tumor margin, as compared to γGlu-HMRG. Additionally, SPiDER-βGal resulted in a high target-to-background ratio due to an intense enhancement within the tumor and those signals lasted up to 60 min after activation (39). These results demonstrated the potential applications of SPiDER-βGal targeted probes for laparotomic and endoscopic detection of primary tumors and metastases.

## Conclusion

The increasing use of genomic and epigenomic information from cancerous tissue is providing new insights into the genetic abnormalities, pathway alterations and target expression, allowing for improved understanding and classification of ovarian cancers. Assimilation of those advances combined with the availability of highly specific probes, chemistry and radiochemistry methods are likely to enhance the potential of theranostic approaches that are already showing promise. Ovarian cancer is also highly suitable for optical imaging applications for surgical guidance. Both preclinical and clinical studies have demonstrated the utility of fluorescent probes for tumor detection during cytoreductive surgery and the development of handheld devices will further increase their use. Although MRI-based studies of ovarian cancer are limited in their scope at this time, the integration of PET/MR scanners could allow for characterization of tumor molecular and metabolic features thus paving the way for imaging and therapeutic guidance by taking advantage of both modalities. These advances on multiple fronts, we believe, are likely to transform our ability to detect, treat and hopefully eradicate ovarian cancer.

## Author Contributions

SN and M-FP wrote the review, and provided approval for publication of the content.

### Conflict of Interest

The authors declare that the research was conducted in the absence of any commercial or financial relationships that could be construed as a potential conflict of interest.
